# Effect of Dendritic Side Groups on the Mobility of Modified Poly(epichlorohydrin) Copolymers

**DOI:** 10.3390/polym13121961

**Published:** 2021-06-13

**Authors:** R. Teruel-Juanes, B. Pascual-Jose, R. Graf, J. A. Reina, M. Giamberini, A. Ribes-Greus

**Affiliations:** 1 Institute of Technology of Materials (ITM), Universitat Politècnica de València (UPV), Camí de Vera s/n, 46022 Valencia, Spain; r.teruel@upvnet.upv.es (R.T.-J.); borpasjo@doctor.upv.es (B.P.-J.); 2 Max Planck Institute for Polymer Research, Ackermannweg 10, Postfach 3148, 55021 Mainz, Germany; graf@mpip-mainz.mpg.de; 3 Department of Analytical Chemistry and Organic Chemistry, Universitat Rovira I Virigili (URV), C/Marcel·lí Domingo s/n, 43007 Tarragona, Spain; joseantonio.reina@urv.cat; 4 Department of Chemical Engineering (DEQ), Universitat Rovira I Virgili (URV), Av. Païssos Catalans 26, 43007 Tarragona, Spain; marta.giamberini@urv.cat

**Keywords:** broadband dielectric spectroscopy, dielectric relaxation spectra, macromolecular cooperativity, segmental dynamics, dendronized liquid crystal membranes, poly(epichlorohydrin), CP-MAS NMR, XRD, DSC

## Abstract

The macromolecular dynamics of dendronized copolymer membranes (PECHs), obtained by chemical modification of poly(epichlorohydrin) with the dendron 3,4,5-tris[4-(n-dodecan-1-yloxy)benzyloxy] benzoate, was investigated. In response to a thermal treatment during membrane preparation, these copolymers show an ability to change their shape, achieve orientation, and slightly crystallize, which was also observed by CP-MAS NMR, XRD, and DSC. The phenomenon was deeply analyzed by dielectric thermal analysis. The dielectric spectra show the influence of several factors such as the number of dendritic side groups, the orientation, their self-assembling dendrons, and the molecular mobility. The dielectric spectra present a sub-Tg dielectric relaxation, labelled as γ, associated with the mobility of the benzyloxy substituent of the dendritic group. This mobility is not related to the percentage of these lateral chains but is somewhat hindered by the orientation of the dendritic groups. Unlike other less complex polymers, the crystallization was dismantled before the appearance of the glass transition (α_Tg_). Only after that, clearing transition (α_Clear_) can be observed. The PECHs were flexible and offered a high free volume, despite presenting a high degree of modifications. However, the molecular mobility is not independent in each phase and the self-assembling dendrons can be eventually fine-tuned according to the percentage of grafted groups.

## 1. Introduction

The dendronized copolymers of poly(epichlorohydrin) (PECH), partially grafted with the dendron 3,4,5-tris[4-(n-dodecan-1-yloxy)benzyloxy]benzoate, are large supramolecular systems. The backbones with their dendritic side groups form a self-assembled structure with arrangement into columnar channels. Their complex behavior is related to the interaction between both parts, and thus, it must be analyzed as a whole [1,2,3,4].

The self-organized dendritic side groups and the backbone in the columnar lattices were discovered and extensively investigated by the group of Percec, which described the properties of these materials as dependent on the shape of the supramolecular architecture [5,6,7].

It is known that the driving forces for the columnar self-assembly of dendronized polymers are given by the aromatic moieties in the dendrons through an exo-recognition process. These polymers can be homeotropically oriented by an external stimulus, due to π–π stacking of aromatic moieties. This evidence was also found in the case of the nanocylinder orientation of a liquid crystal block copolymer film, based on poly(ethylene oxide) and a polymethacrylate bearing azobenzene mesogenic side chains. Due to these properties, the membranes prepared with these materials show special characteristics that allow them to selectively transport charges, gases, and liquids. In particular, the membranes based on dendronized PECH have demonstrated promising properties in terms of proton transport and selectivity [8,9,10,11,12].

Nevertheless, the self-organized columnar structure has several limitations, resulting from the complexity of its molecular design, which restricts the number of possible applications of these materials. To obtain a certain performance, a specific orientation and size of the columns are required, as well as the combination of both order and mobility on a macroscopic level. In this sense, determining the relationship between the structure and the mobility of the large supramolecular systems is essential to design membranes that respond to the entailed properties.

Dielectric spectroscopy is a suitable technique to determine the reorientation of the permanent dipoles present on the side chains and on the backbone of polymeric materials, which manifest as molecular relaxations. Considering the response to an electrical perturbation field over a wide frequency range at different temperatures, the dielectric spectrum of these copolymers is obtained, and it provides information on the dynamics of large supramolecular systems [13,14].

Differential scanning calorimetry (DSC), X-ray diffraction (XRD), and ^13^C cross polarization magic-angle spinning (CP-MAS) NMR contribute to characterize these materials, as far as their structure and tendency to crystallize is concerned.

In a recently published manuscript [15], the dielectric properties of unoriented and oriented membranes, based on dendronized polymers and copolymers obtained by chemical modification of poly[2-(aziridin-1-yl) ethanol] (PAZE) with the dendron 3,4,5-tris[4-(n-dodecan-1-yloxy)benzyloxy]benzoate, were analyzed. This study confirms changes in the molecular mobility of the dendritic side groups and the stability of the nano-channels with the orientation and the addition of dendritic groups. The dynamic fragility increases, with the increase in the grafting of the dendritic groups to the backbone structure, as well as a significantly weaker columnar structure. Nevertheless, the orientation procedure by means of an external stimulus, such as the thermal treatment during membrane preparation, increases the glass transition temperature and confirms the stabilization of the dendritic structure.

This work analyses the dielectric relaxation spectra of two copolymers, oriented and unoriented poly(epichlorohydrin) (PECH), with different proportions of mesogenic side groups, in order to understand the role of the backbone in self-assembled structures and the interaction between the side groups. These copolymers have the same mesogenic side groups, such as the PAZE copolymers analyzed in the previous work, but differ in the presence of the basic oxygen atoms instead of nitrogen in their backbones.

To predict the final properties of these functionalized copolymers, two factors must be considered, chain flexibility and structure regularity. Thus, it is desirable to establish whether the properties of these supramolecular systems may be tuned on the basis of either applying a thermal treatment or changing the number of grafted dendrons.

## 2. Materials and Methods

### 2.1. Materials and Membrane Preparation

Commercial poly(epichlorohydrin) (PECH) was purchased from Aldrich (St. Louis, Missouri, USA). Weight-average molecular weight (M_w_) was 8.52 × 10^5^ and the number-average molecular weight was (M_n_) 3.2 × 10^5^, which were determined by size exclusion chromatography-multi-angle laser light scattering (SEC-MALLS). The copolymers were obtained by grafting the net poly(epichlorohydrin) with 3,4,5-tris[4-(n-dodecan-1-yloxy)benzyloxy]benzoate.

Copolymers have been labeled PECH80 and PECH40, where 80 and 40 represent the degree of modification of the net polyethers, PECH. The routes of synthesis and the characteristic differences are reported elsewhere [3,16,17].

**Figure 1:**
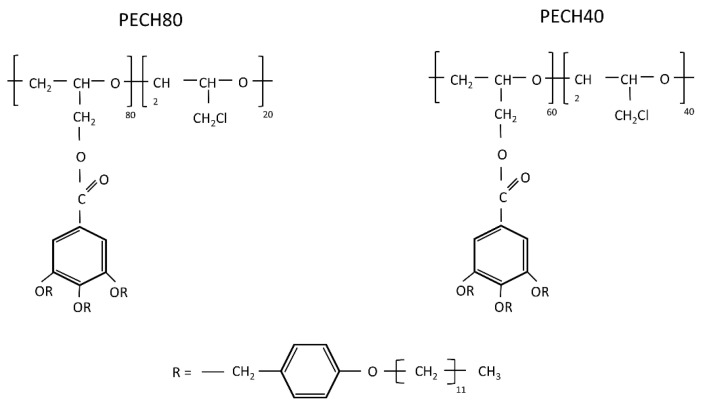
Molecular structure of the PECH copolymers and the dendron 3,4,5-tris[4-(n-dodecan-1-yloxy)benzyloxy]benzoate.

The final chemical structure is schematized in Figure 1.

Membranes were prepared out of these polymers using phase inversion precipitation onto a Teflon^®^ substrate. Homeotropic orientation of the LC columns was obtained by following the thermal treatment previously reported [3,17]. This columnar orientation is perpendicular to the membrane surface and improves the formation of ionic channels, which allows efficient cation transport. The thermal treatment consists of heating the obtained membranes above the copolymer clearing temperature on Teflon^®^ support with a Linkam TP92 hot stage and keeping them at this temperature for ten minutes. Subsequently, they were slowly cooled (0.5 °C·min^−1^) to room temperature and then separated from the Teflon^®^ support. Oriented copolymers are labeled as PECH80-O and PECH40-O.

### 2.2. Physico-Chemical Characterization

#### 2.2.1. Differential Scanning Calorimetry (DSC)

Calorimetric studies of dendronized polymers were performed in aluminum standard 40 mL crucibles without pin (ME-26763) with a Mettler DSC822e thermal analyzer (Mettler Toledo, Columbus, OH, USA) at the heating rate of 10 K/min using about 5 mg of sample, nitrogen as a purge gas (100 mL/min) and liquid nitrogen for the cooling system. The equipment was previously calibrated with indium (429.7 K) and zinc (692.6 K) pearls.

#### 2.2.2. Polarized Optical Microscopy (POM)

Clearing temperatures were estimated using polarized optical microscopy (POM); textures of the samples were observed with an Axiolab Zeiss optical microscope (Carl Zeiss Microscopy, Oberkochen, Germany) equipped with a Linkam TP92 hot stage (Tadworth, UK). An image (Figure S1) of the PECH40 can be found in the Supplementary Materials.

#### 2.2.3. Solid-State Nuclear Magnetic Resonance (NMR) Spectroscopy

For all samples ^1^H MAS and ^13^C CP-MAS spectra have been recorded in a temperature range from 250 K to 350 K. The measurements have been performed at 25 kHz MAS and 850 MHz ^1^H Larmor frequency using a Bruker Avance III console (Bruker Corporation, Billerica, Massachusetts, USA) and a commercial double-resonance MAS probe supporting zirconia rotors with 2.5 mm outer diameter. The RF power levels on ^1^H and ^13^C have been adjusted to an RF nutation frequency of 100 kHz, corresponding to a 90° pulse length of 2.5 µs. The CP-MAS spectra have been acquired with a CP contact pulse length of 1 ms and 2048 transients, using the SPINAL64 decoupling scheme during acquisition. The adjusted VT gas temperatures have been corrected for frictional heating under fast MAS conditions using the known temperature-dependent chemical shift of lead nitrate so that the given temperature values reflect the actual sample temperature under the chosen MAS conditions.

#### 2.2.4. X-ray Diffraction (XRD)

X-ray diffraction measurements were performed with a Siemens (Siemens AG, München, Germany) D5000 diffractometer (Bragg–Brentano parafocusing geometry and vertical θ-θ goniometer) fitted with a curved graphite diffracted-beam monochromator, incident and diffracted-beam Soller slits, a 0.06° receiving slit and scintillation counter as a detector. Samples were dissolved in a few drops of THF, placed directly onto a low background Si(510) sample holder for reflection analysis and then the solvent was evaporated before analysis. The X-ray diffractometer was operated at 40 kV and 30 mA to generate CuKα radiation. The angular 2θ diffraction range was between 1 and 40°. The data were collected with an angular step of 0.03° at 6 s per step.

#### 2.2.5. Dielectric Thermal Analysis (DETA) Assessment

The dielectric spectra were measured under isothermal conditions in the frequency range of *f* = 10^−2^–10^7^ Hz, at temperatures between 123 K and 393 K, with dielectric thermal analysis equipment (DETA-Novocontrol Technologies GmbH & Co. KG, Hundsangen, Germany). To avoid any interference caused by electrode polarization, a PTFE film was inserted between the sample and the electrode [15,18,19]. The intermolecular relaxations were characterized in terms of dynamic fragility, free volume, and thermal expansion coefficients as has been carried out elsewhere [20,21,22,23,24,25].

## 3. Results

### 3.1. Morphological and Calorimetric Characterization

**Figure 2:**
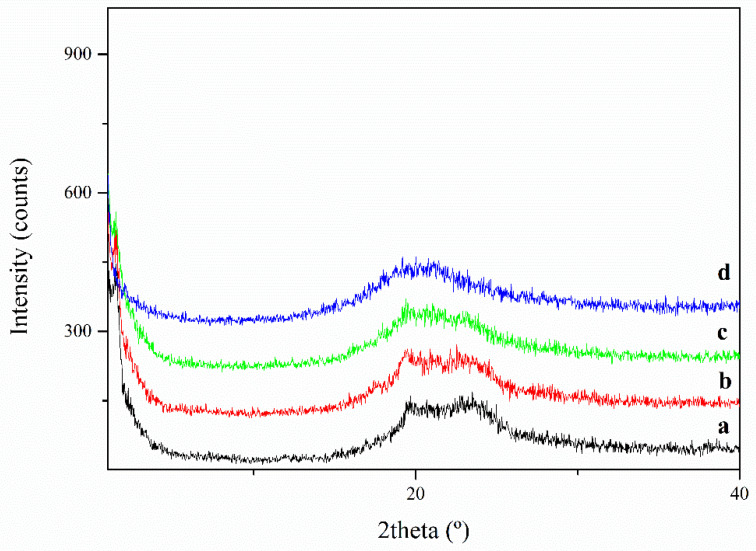
XRD diffraction pattern of PECH40 at (**a**) 223 K; (**b**) 283 K; (**c**) 297 K; (**d**) 383 K.

The presence of crystallinity and liquid crystalline organization in the copolymers PECH40 and PECH80 was analyzed by X-ray diffraction (XRD). The measurements were carried out at different temperatures and the XRD diffractograms were recorded according to the following schedule, designed on the basis of the DSC evidence shown below: (i) start at 297 K; (ii) cooling 1 K/min to 223 K; (iii) heating 10 K/min to 283 K; (iv) heating 10 K/min to 383 K. Figure 2 shows the corresponding diffractograms of PECH40 at the temperature of 297 K. A reflection at a low angle (around 2θ = 1.5°) was observed, which may be assigned to the columnar mesophase. Figure 2 also recorded the diffractogram at 223 K. It puts into more evidence this reflection at 2θ = 1.6°, corresponding to d = 55.2 Å; besides, a very small crystalline reflection at 2θ = 19.8° can be foreseen. Moreover, the sharpening and asymmetry of the region around 20° also suggest the presence of some crystallinity. These small reflections were still evident when the experiment was performed at 283 K. When the sample was further analyzed at 383 K, only the broad halo around 2θ = 20° could be observed. This result evidences the existence of crystalline domains below 300 K and a columnar mesophase up to about 353 K.

**Figure 3:**
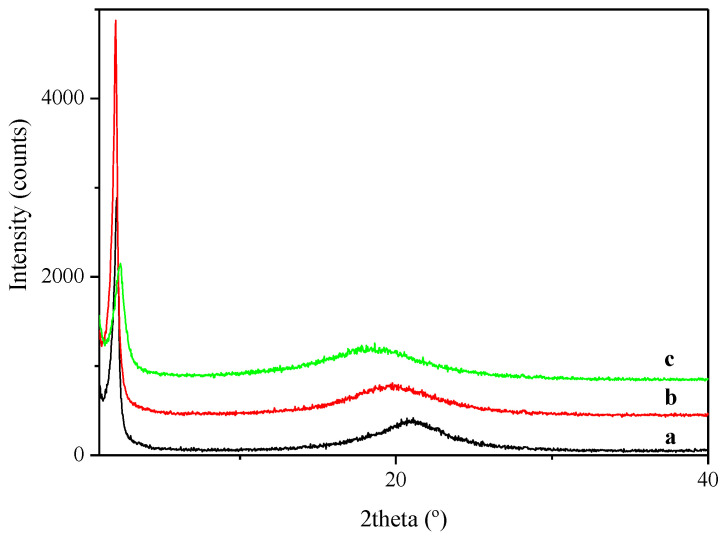
XRD diffraction pattern of PECH80 at (**a**) 223 K; (**b**) 303 K; (**c**) 433 K.

Similar to PECH40, in the case of PECH80, XRD was recorded according to the following schedule: (i) start at 303 K; (ii) cooling 1 K/min to 223 K; (iii) heating 10 K/min to 433 K. Figure 3 shows the XRD patterns recorded at three selected temperatures. At all the investigated temperatures, one can only see a halo around 2θ = 20° and a reflection around 2θ = 2°, characteristic of the LC columnar phase.

**Figure 4:**
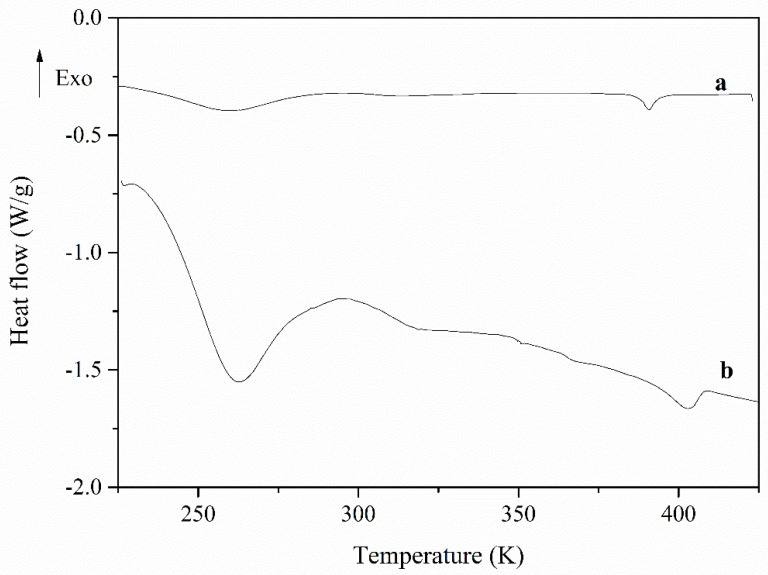
DSC second heating scan (rate: 10 K/min) of PECH40 (**a**) and PECH80 (**b**).

On the other hand, Figure 4 displays the calorimetric thermograms corresponding to the second heating scan of the PECH40 and PECH80 copolymers, performed at a 10 K/min heating rate. In both cases, it is possible to distinguish at least two prominent endotherms, the first one around 262 K and the other at 402 K. Between both, a small shoulder can be observed around 310 K. The combination of XRD and calorimetric analyses leads to the following conclusions: The former endotherm can be related to the melting of a small crystalline portion formed, while the latter relates to the clearing transition, both associated with the presence of the lateral mesogenic chains. The second shoulder could be assigned to the glass transition or/and the melting of another crystalline phase [26].

Table 1 reports the following important calorimetric features: melting temperature T_m_; glass transition temperature T_g_; clearing temperature T_c_; melting enthalpy Δ*H_m_*; and clearing enthalpy ΔH_c_ of PECH40 and PECH80.

### 3.2. Analysis of the Dielectric Spectra

The dielectric behavior of unoriented (PECH 40, PECH 80) and oriented (PECH 40-O, PECH 80-O) copolymers showed dielectric relaxations for the whole range of temperatures and frequencies, which are related to macromolecular motions in a wider temperature range. The dielectric relaxation spectra of these copolymers were analyzed in terms of the real (ε′) and imaginary (ε″) parts of the complex dielectric permittivity (ε*), and tan δ in the temperature range between 110 K and 400 K, in the frequency interval of 10^−2^ to 10^7^ Hz. The dielectric relaxations plotted in terms of the loss factor, ε″, were initially deconvoluted following the Charlesworth method. To account for the additive character of the dielectric response, the data were fitted by adding up as many Havriliak–Negami (HN) functions as relaxation processes necessary to adjust all the values for each temperature. The HN function for *ε*″ is shown in Equation (1) [27,28,29,30].

(1) $$\varepsilon^{*}\left(w\right)-\varepsilon_{\infty }=\;\underset{k}{\sum }Im\left[\frac{\Delta \varepsilon }{{\left(1+{\left(iw\tau_{HN_{k}}\right)}^{\alpha_{k}}\right)}^{\beta_{k}}}\right]$$

where *α_k_* and *β_k_* are parameters corresponding to the width and asymmetry of the relaxation time distributions, respectively, *τ_HNk_* is the Havriliak–Negami relaxation time, and the sub-index *k* represents the number of the individual contributions (*k* = 1 to 3, depending on the complexity of the *ε*″ curve at any temperature). The following parameters: the relaxation time (τ), the values of the dielectric intensity or relaxation strength (Δ*ε*), and a_HN_, b_HN_ Havriliak–Negami parameters were assessed and compared between both the oriented and unoriented copolymers.

The relaxation time (*τ*), a_HN_, b_HN_ Havriliak–Negami parameters, and the values of the dielectric intensity or relaxation strength (Δ*ε*), were assessed and compared between both the oriented and unoriented copolymers.

(2) $$\Delta \varepsilon =\varepsilon_{s}-\varepsilon_{\infty }$$

with $\varepsilon_{s}=\varepsilon^{\prime }$ at f→0 and $\varepsilon_{\infty }=\varepsilon^{\prime }$ at f→∞.

**Figure 5:**
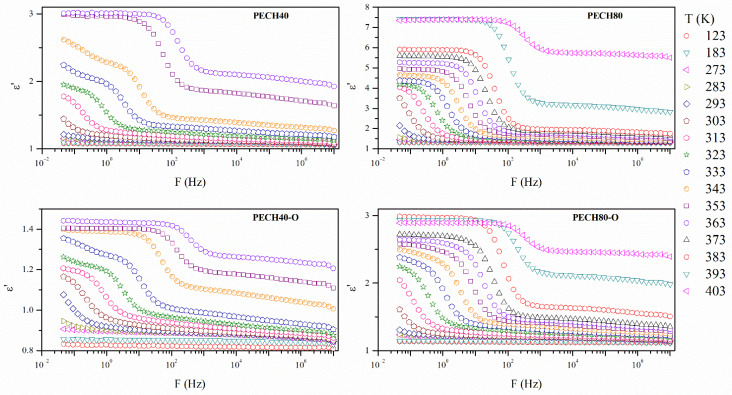
Isothermal curves (in K) of the real part of the dielectric permittivity of oriented and unoriented PECH copolymers.

**Figure 6:**
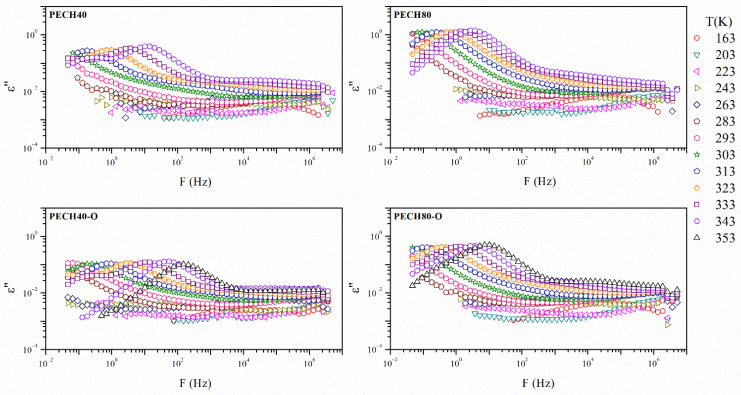
The imaginary part of the dielectric permittivity of oriented and unoriented PECH copolymers.

Figure 5 and Figure 6 display the isothermal curves of the real ε’ and imaginary *ε*″ parts of the complex dielectric permittivity ε* for all the unoriented and oriented samples. All the analyzed isothermal curves display a similar pattern, one plateau, followed by a decreasing step, which is especially evident for the PECH80 copolymer.

**Figure 7:**
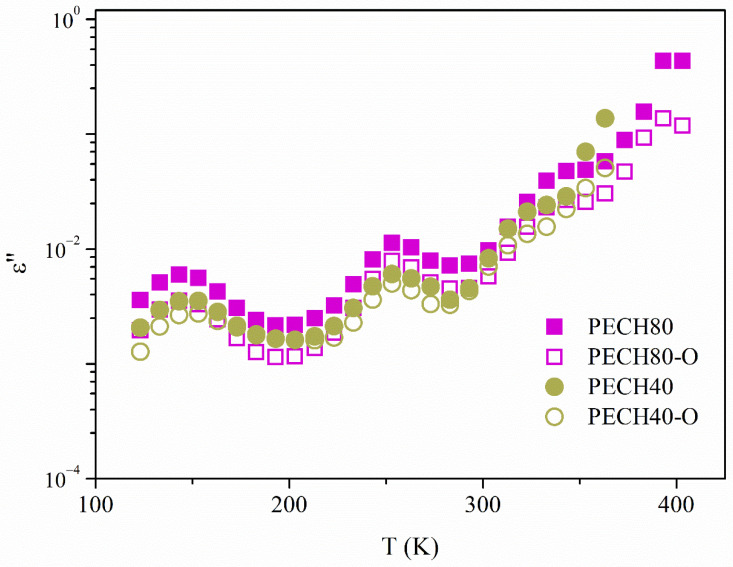
Comparative isochrones curves at 1 kHz of the imaginary part of dielectric permittivity of unoriented and oriented PECH80, PECH40.

Besides, Figure 7 displays the evolution with the temperatures of the isochronal loss tan δ curves of all the unoriented and oriented samples.

In general terms, Figure 6 and Figure 7 exhibit a complex spectrum with at least two broad and complex dielectric relaxations, which may be the result of overlapped molecular mobilities. These dielectric relaxations have been labeled as γ and α with increasing temperature, and may be related to the different molecular motions of the benzyloxy group of the side dendrons, glass transition (α_Tg_), and clearing of the mesophase (α_Clear_), respectively.

Different factors must be taken into account to compare and analyze the dielectric spectrum of these systems, such as chain flexibility, structure regularity, and modification extent. The former can be responsible for an easier, but not necessarily stable, arrangement of the polymers into compact, ordered, or pseudo-ordered domains. In addition, the presence of side dendrons gives rise to an exo-recognition process, which favors polymer self-organization. Structure regularity is crucial for the formation of a stable crystalline phase. Nevertheless, the dielectric relaxation spectrum does not show large differences between the oriented and unoriented samples. The thermal orientation affected similarly the PECH copolymers.

The modification degree of the copolymers affected their behavior. In previous studies [7], it has been reported that the polymer backbone has no meaningful influence on the columnar self-assembly of dendronized polymers in the orientation of the polymeric columns perpendicular to the membrane surface. In general, the aromatic moieties are responsible for the self-assembly process of the dendrons and induce a helical arrangement of the main chain [31].

However, the higher the presence of liquid crystalline dendritic units in the structure was, the higher the polarization effect was, as the values of *ε*’ showed. The orientation decreased the values of the *ε*’ of the PECH copolymers, probably as a consequence of the increase in the crystalline content produced during thermal orientation.

Table 2 shows the dielectric strength below and above the glass transition of the unoriented and oriented PECH80, PECH40. As expected, the values increase with the temperature. The dielectric strength slightly decreased in the PECH copolymers when the orientation was induced by the thermal treatment.

**Figure 8:**
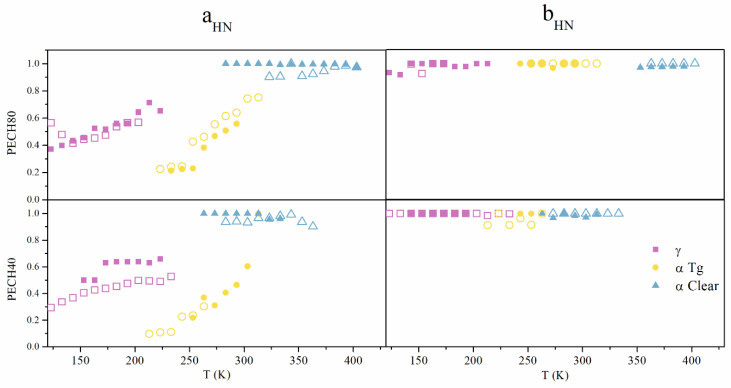
Comparative isochrones curves at 1 kHz of the imaginary part of dielectric permittivity of unoriented and oriented PECH80, PECH40.

The Havriliak–Negami shape a_HN_, b_HN_ parameters, which were calculated for each system below and above T_g_, are shown in Figure 8. The relationship between both parameters with temperature is very different. While b_HN_ parameters corresponding to all relaxations were very close to the unity, independently of temperature, a_HN_ parameters, as expected, increased with the temperature and lie in the range 0.8−0.9 at higher temperatures. This fact suggests a Cole−Cole behavior at high temperatures that come close to a semicircle in the low-frequency region. The orientation induced by heat treatment slightly reduced these values at γ relaxation and increased in the α relaxation. The crystalline organization of chains could be promoting a rather narrow distribution of relaxation times.

#### 3.2.1. Macromolecular Origin and Thermal Activation of the γ Relaxation

**Figure 9:**
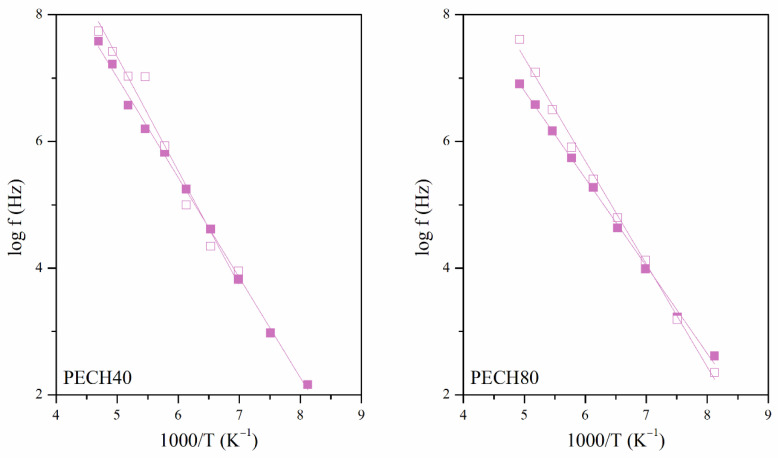
Arrhenius maps of the γ relaxations of unoriented (full symbol) and oriented (hollow symbol) PECH80 and PECH 40 copolymers.

The γ relaxation was located at low temperatures between 132 K and 128 K at a frequency of 1 kHz. Figure 9 plots the Arrhenius map of the oriented and unoriented copolymers. This figure shows a linear behavior in the *log f* vs. *T*^−1^, which means a temperature-frequency dependence for the maxima of loss factor isochrones, and, consequently, the thermal activation is governed by an Arrhenian-like function.

(3) $$f_{max}=f_{0}\cdot exp\left(\frac{-E_{a}}{RT}\right)\;\;\;\;$$

where *f_max_* = 1/*τ_max_* is the frequency corresponding to the maxima in the relaxation peak (thus the reciprocal of the relaxation time, *τ_max_*), *T* is the temperature in Kelvin, *R* is the gas constant of 8.31 J·mol^−1^·K^−1^, *E_a_* is the activation energy, and *f*_0_ is a pre-exponential term.

As Table 3 shows, the apparent activation energies *E_a_* lie between 25 and 35 kJ·mol^−1^. These low values are characteristic of orientational changes of small angles concerning the longitudinal backbone of the copolymer. These motions were ascribed to the local mobility of the local secondary phenyl-aliphatic chain of the dendritic liquid crystal. The increase in the grafting degree has a slight increase in the apparent activation energies. The orientation during the preparation of the membrane also shifted the position of the maxima to higher temperatures. Polymer orientation determines a more ordered arrangement of polymer structure; therefore, more energy is needed to activate the dielectric relaxations in PECH copolymers. These results agree with those obtained from similar copolymers and confirm that orientation decreases the molecular motion of the lateral chains and, accordingly, the permittivity of the membranes [15].

#### 3.2.2. Macromolecular Origin and Thermal Activation of the α Relaxation

At higher temperatures, close to the glass transition, both of the PECHs copolymers showed complex dielectric spectra, as is usually expected for dielectric relaxations of polymers turning from the glassy to the rubbery state, which high viscosity eases large-scale polarization effects. A high-intensity relaxation, constituted by two or more phenomena very close, was observed. These can be related to motions in both the side chains and the main chain, which makes their characterization difficult.

**Figure 10:**
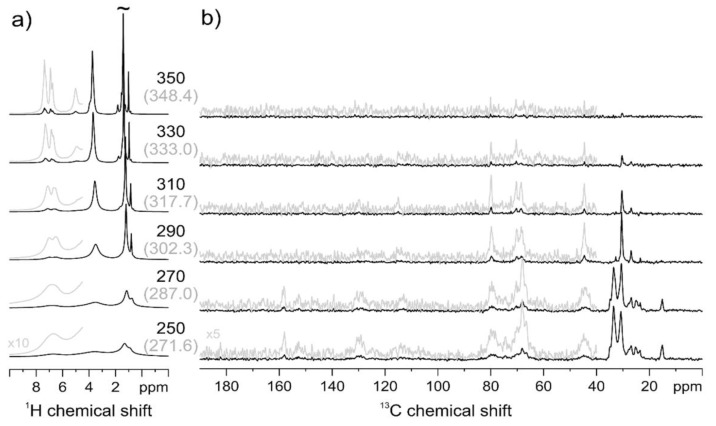
PECH40 ^1^H MAS (**a**) and ^13^C CP-MAS (**b**) NMR spectra at variable temperature. In brackets, the temperature values corrected for frictional heating under fast MAS conditions are reported.

To elucidate the molecular motions that are the origin of these relaxations, the ^1^H MAS NMR and ^13^CP-MAS NMR spectra were recorded at different temperatures, and allowed us to distinguish more mobile and more rigid sites of the copolymers PECH40 and PECH80. Figure 10a gives the temperature-dependent ^1^H MAS NMR spectra of PECH40. Over the whole temperature range from 272 to 348 K, a gradual narrowing of all signals is observed. At all temperatures, the best spectral resolution is observed for the aliphatic sites of the outer side chains around 1 ppm, suggesting that the increasing molecular mobility with increasing temperature develops from the end of the aliphatic side chains. The temperature-dependent ^13^C CP-MAS NMR measurements shown in Figure 10b indicate a gradual simultaneous fading of the different signals of the side chains and along the polymer backbone, and thus increasing molecular mobility when the temperature increases. These results are in agreement with the dielectric result and confirm that motions of both the side chains and the main chain are in the origin of two or more overlapping α relaxation processes.

In PECH40, the aliphatic side chains are highly crystalline at low temperatures, as indicated by the signal at 34 ppm in the ^13^C CP MAS spectrum. Between 272 K and 302 K, the aliphatic side chains melt, as can be seen from the disappearance of the ^13^C NMR signal at 34 ppm as well as from the sudden decrease in the line width of the aliphatic signals in the ^1^H MAS spectrum, where the CH_3_ group at 0.8 ppm and the CH_2_ signals at 1.2 ppm are then clearly resolved. Therefore, these results corroborate that the first peak shown by DSC can be reasonably attributed to a side-chain melting. Above 333 K the motion of the molecules becomes fully isotropic, which leads to the loss of all CP-MAS signals. The very narrow signals in the ^1^H MAS spectrum at T = 350 K indicate a very high local mobility of all sites (polymer backbone, aromatic linker, and aliphatic side chains) of the polymer. The simultaneous fading of all signals indicates that there is only a minor mobility gradient between the different sites, as suggested from ^1^H MAS NMR.

**Figure 11:**
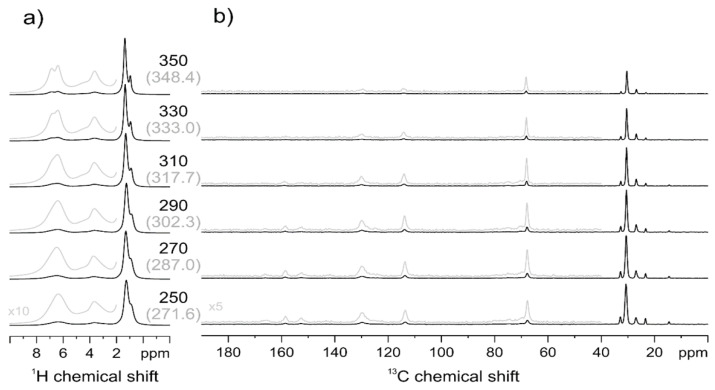
PECH80 ^1^H MAS (**a**) and ^13^C CP-MAS (**b**) NMR spectra at variable temperature. In brackets, the temperature values corrected for frictional heating under fast MAS conditions are reported.

The temperature-dependent ^1^H MAS NMR spectra of PECH80 are given in Figure 11a, which shows a gradual line narrowing of all signals, very similar to the behavior of PECH40.

Comparing the individual line shapes, similar spectral line shapes for the sample PECH80 are shifted towards higher temperatures by approximately 40–50 K, and in particular, the ^1^H signals of the aliphatic side chains remain significantly broader even at the highest temperature accessible to our NMR setup, which is still significantly below the isotropization temperature of 403 K determined by DSC. The temperature-dependent ^13^C CP-MAS spectra given in Figure 11b confirm the gradual increase in mobility. Remarkably, the decay of the signal intensity with increasing temperature appears to be non-uniform for the different sites. The signal intensities of the aromatic site seem to fade more rapidly with increasing temperature compared to the methoxy and aliphatic sites. However, the interpretation of this finding is very difficult as the aromatic CH bonds have an orientation of 60° degrees to the 1,4 axis of para-substituted phenylene rings, which leads to a much efficient motional averaging of CH dipolar couplings compared to the averaged orientations of methoxy or methylene groups. It should be pointed out that a significant motion, induced fading of the CP-MAS NMR signals in PECH80 occurs only above the glass transition temperature observed at 305 K in DSC. The glass transition is kinetic and so the temperature at which it occurs depends on the frequency, and the Williams–Landel–Ferry (WLF) equation predicts that T_g_ will change 6°–7° per decade of frequency [32].

Within the experimental error, the relation between the temperature and frequency follows the WLF equation even at the very high frequencies involved in NMR relaxation [33]. The DSC observation range is in the 1 Hz regime, whereas the NMR spectra allow the observation of molecular motions in the 10–100 kHz frequency regime; therefore, we could expect a shift of T_g_ approximately 30 K in the ^13^CP-MAS and ^1^H MAS NMR spectra.

In comparison to PECH40, where the CP-MAS signals fade completely in a narrow temperature range, the liquid crystalline order in PECH80 is sufficient to retain strong residual heteronuclear dipolar couplings for an efficient CP polarization transfer. This nicely coincides with the significantly higher enthalpy of PECH80 at the isotropization transition compared to PECH40. Therefore, higher modification degrees determine an increase in polymer rigidity, as it can be reasonably predicted; PECH80 contains more mesogenic units and has a higher clearing temperature compared to the less modified and less ordered PECH40. It should be remembered that starting PECH material is completely amorphous, and therefore the presence of the mesogenic side groups is responsible for inducing a hierarchical self-organization in the modified polymers through an exo-recognition process.

Therefore, according to the above calorimetric results, the first detected endotherm put into evidence the existence of a slight crystalline portion in these copolymers, which might be modified by the thermal treatment reported in the experimental part.

From calorimetric data, we could roughly estimate the crystallinity degree xc from the following equation [34]:

(4) $$x_{c}=\frac{\Delta H_{m}}{\Delta H_{m0}}$$

where Δ*H_m_* is the observed heat of melting per mesogen and Δ*H_m_*_0_ is the heat corresponding to 100% crystallinity, and it is equal to *C + K·n*; *K* is the constant heat of melting per mol methylene group, *n* is the number of methylene groups in the chain, and C takes into account the contribution of the terminal groups. Under the assumption that the crystalline side chains adopt the orthorhombic, all-anti zigzag conformation, we can consider the constant values found for polyethylene as *K* = 3.97 kJ/mol and *C* = −12.29 kJ/mol. In this way, the degree of crystallinity was estimated as 12% for PECH40 and 10% for PECH80 [35].

In previous papers [1,15], the thermal process gave satisfactory orientation when the membrane is lying on both a hydrophilic or a hydrophobic substrate (treated glass or Teflon^®^ sheet, respectively), suggesting that the hierarchical structures in these copolymers tend to grow down from the top surface. This can be attributed to the dendrons anchoring to the air interface; in other words, the aliphatic tails of the dendrons, which possess higher mobility and low surface energy, would drive the dendrons towards the air interface over the main chain components. This evidence was also found in the case of the nanocylinder orientation of a liquid crystal block copolymer based on poly(ethylene oxide) and a polymethacrylate, also bearing azobenzene mesogen side chains [36].

In this same zone, and probably with values of temperatures in the surroundings to the glass transition to slightly higher temperatures, the clearing phenomenon also takes place. It is a supramolecular phenomenon that corresponds to the disassembling of the polymeric columns, which therefore provokes the disappearance of the liquid crystalline order. The clearing is a typical transition of thermotropic liquid crystals, based on the predominance of thermal motions over the weak interactions between the dipole-type molecules or dispersion forces that determine the arrangement into a liquid crystalline phase. These interactions are strong enough to maintain associations between molecules in a preferred orientation; however, molecules keep their freedom to move, since they are not covalently bound. In the case of the systems under investigation, the grafted tapered moieties form layers with their neighbors that are arranged at different angles, giving a liquid crystal with a helical arrangement. Thus, these moieties are placed parallel, but at the same time they can move one another along their axes. When the temperature is raised above the clearing point, thermal motions prevail over dispersion forces, and the polymeric material undergoes a first-order transition and turns isotropic.

In DETA analysis, the α_Clear_ relaxation appeared at temperatures between 315 and 337 K, according to the macromolecular investigations described in Table 2.

In agreement with the above results, the complex relaxation spectrum of unoriented and oriented PECH40 and PECH80 copolymers were analyzed with the following two relevant relaxations: α_Tg_ relaxation, related to the glass transition, and the other one being α_Clear_ relaxation, related to the clearing transition. However, the DSC observation shown in Table 1 suggests the existence of a third process at lower temperatures than the glass transition and overlapped to it, which can be related to precursor molecular motions of the melting of a certain crystalline portion induced by the presence of the lateral side chains.

Furthermore, the melting of these crystalline entities would occur before the co-axial crank-shaft motion that gives rise to the glass transition. Nevertheless, in the set of all relaxations, the one associated with the precursor motion of the fusion of this crystalline zone could not be observed. When melting occurs, probably it is completely masked by the relaxation associated with the glass transition; nevertheless, as it occurs at slightly lower temperatures, its presence may only displace the maximum temperature value of the α_Tg_ peak. Thus, the relaxation related to this motion cannot be observed.

The side-chain crystallinity, therefore, seems to be the main factor to influence the mobility of the main chain and therefore α_Tg_ relaxation; on the other hand, the orientation during membrane preparation did not affect the temperature for this relaxation since, when the cooperative motion of the main chain begins, the crystallinity of the side chains has already disappeared. The same occurs with the relaxation related to polymer clearing, i.e., α_Clear_, the orientation did not seem to affect the clearing process.

**Figure 12:**
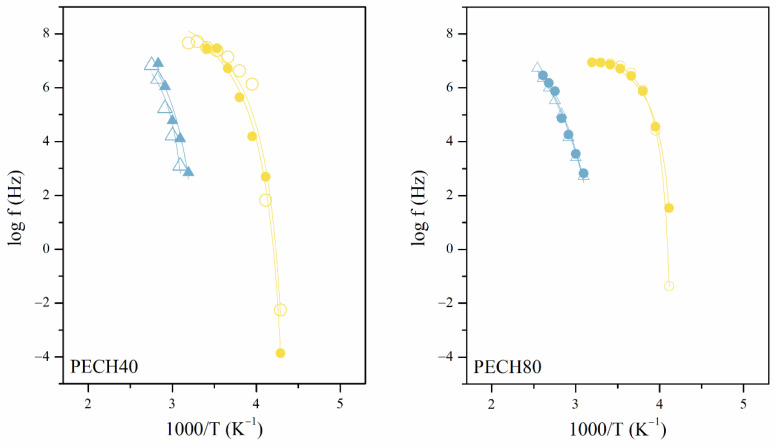
Arrhenius maps of the α_Tg_ and α_Clear_ relaxations of unoriented (full symbol) and oriented (hollow symbol) PECH80 and PECH40 copolymers.

To confirm the hypothesis of macromolecular cooperativeness, Figure 12 displays the Arrhenius plots of oriented and unoriented copolymers. The two relaxations were observed with a non-linear dependence between *log f* vs. *T*^−1^ representative of VFTH-like thermal activations, inherent of intermolecular segmental motions.

These intermolecular relaxations were characterized in terms of the dynamic fragility, free volume, and thermal expansion coefficient, as has been carried out elsewhere. Table 4 displays the VFTH parameters of α_Tg_ relaxation for unoriented and oriented PECH80 and PECH40 copolymers. Thus, the relationship between the relaxation time and the temperature was calculated by Equation (5) [15,21,22,23,25,37].

(5) $$f_{max}=f_{0}\cdot exp\left(\frac{-B}{T-T_{0}}\right)$$

where *f_max_* = 1/*τ_max_* is the frequency corresponding to the maxima in the relaxation peak (thus the reciprocal of the relaxation time, *τ_max_*), *T* is the temperature in Kelvin, *R* is the gas constant of 8.31 J·mol^−1^·K^−1^, *E_a_* is the activation energy, *f*_0_ is a pre-exponential term, *B* is related to the apparent activation energy,

(6) B=EaR(1−T0T)2

and *T*_0_ is the Vogel temperature, below which polymeric segments become immobile. *B* is also related to the parameter *D*,

*B* = *D*·*T_K_* (7)

which is correlated to the topology of the theoretical potential energy surface of the system, where fragile systems (*D* ≤ 6) present a high density of minimum energy, contrarily to strong systems (*D* ≥ 15), which present lower density.

The values of these parameters in Table 4 indicate that the orientation during preparation did not have much effect on the temperature of both relaxations, as previously explained.

Nevertheless, the fact that the melting occurs before the glass transition is not a common phenomenon and reflects the complexity of the structure of these copolymers. In a single-phase system, glass transitions must happen below the melting temperature. When amorphous and crystalline phases coexist, as is the case of polyethylene, it was reported that the α relaxation is associated with the crystalline phase and involves softening or deformation of the amorphous regions or splits it into two components, related to the lamellar thicknesses of crystals at the amorphous–crystalline interface [38,39,40,41,42].

For poly(L-lactic acid), dielectric loss curves put into evidence an α-process of the bulk-like amorphous phase, and an α-process of the constrained amorphous portion, in which the segmental motions are affected by the steric constraint exerted by the crystalline environment; this α-constrained relaxation process shows the usual curvature of cooperative relaxations in the Arrhenius maps [25,43].

However, for PECHs copolymers the relaxation process of the amorphous portions is somewhat constrained by the crystalline order of the dendritic lateral groups, and only when the crystalline order disappears upon melting does the molecular motion of the backbone start.

Thus, the modification degree is the most relevant factor for α_Tg_ and α_Clear_ relaxations of PECH copolymers. According to the above and elsewhere DSC results, the presence of the dendritic side chains forces the copolymers to adopt a specific spatial situation and constrains their steric influence; this is also probably promoted by the high molecular weight of these copolymers [1,2,44,45].

Concerning the α_Tg_, the chain segment relaxes when the available free volume is higher than a critical volume, which is defined by the cooperative length of the segments. Thus, the values of the dynamic fragility parameter *D* are low enough to correspond, as Angell classification, as fragile systems. The dynamic fragility parameter *D* is lower in the oriented and unoriented PECH80 copolymers than in the PECH40 ones. This is in line with the high values of the free-volume coefficients *Φ*/*B* and the thermal expansion coefficients *α_f_*. However, T_VFTH_ (K) is higher in the PECH80 copolymers, which indicates higher stability of these copolymers above the glass transition. Thus, PECH80 copolymers maintain a self-organized columnar structure more stably than PECH40 ones at temperatures below the glass transition [20,23,46,47].

Nevertheless, when the systems approach the glass transition, the lower values of the parameter *D* in the PECH80 copolymers indicate the growth of macromolecular rearrangements, which provoke the crank-shaft motion and promote a quick loosening of the rigid glassy structure. This heterogeneous behavior near the glass transition and below the clearing transition indicate that some side chains are already quite mobile, whereas others are still very rigid. This equilibrium between the mobility and stabilization of the dendritic chains allows turning these polymers into efficient membranes for ionic transport applications. These results are in agreement with the behavior of dendronized polymers and copolymers obtained by the chemical modification of poly[2-(aziridin-1-yl) ethanol] (PAZE), with the same dendron 3,4,5-tris[4-(n-dodecan-1-yloxy)benzyloxy]benzoate analyzed elsewhere [15]. These copolymers with lower grafted dendritic groups maintain better the locally ordered structure near the glass transition than the polymer completely grafted.

Near α_Tg_ relaxation, the phenomenon of clearing also took place. The α_Clear_ relaxation is related to a precursor motion to the disassembling of the polymeric columns. The clearing is a supramolecular phenomenon that provokes the disappearance of the thermotropic liquid crystals order, as this temperature rises, the order of the system decreases, and a liquid crystal phase is converted into an isotropic liquid. In the PECH copolymers, the clearing temperature appears between 353 K and 405 K, according to the DSC results displayed in Table 1. The molecules get their freedom due to the predominance of thermal motions over the weak dipolar interactions between molecules.

As the Arrhenius plots in Figure 12 indicate, the α_Clear_ relaxation is an intermolecular motion and could be therefore explained by a VFTH-like function. Table 5 gathers the results of fitting the thermal activation of the relaxation times for oriented and unoriented copolymers. Despite this, only the highest regression parameters to the VFTH-like function have been considered. From these parameters, it can be deduced that orientation during the preparation of the samples did not have any effect on this relaxation process, as expected.

However, the grafted tapered moieties form layers with their neighbors that are arranged at different angles, giving a liquid crystal with a helical arrangement. Thus, these moieties are placed parallel, but at the same time, they can move past each other along their axes. For the α_Clear_ relaxation, the parameter *D* exhibits higher values for both the oriented and unoriented PECH80 copolymers than for the PECH40 ones. Besides, PECH40 has a T_VFTH_ temperature related to the clearing relaxation higher than PECH80 copolymers. These results indicate that dispersion forces prevail when the number of the grafted dendritic group increases. Thus, the polymeric material undergoes a first-order transition and turns isotropic faster.

## 4. Conclusions

The dendronized copolymers, obtained by the chemical modification of poly(epichlorohydrin) (PECH) with the dendron 3,4,5-tris[4-(n-dodecan-1-yloxy)benzyloxy]benzoate, partially organize into a crystalline structure and a liquid crystal mesophase. The thermal treatment applied for membrane orientation enhances nano-crystalline structures.

The dynamic mobility analyses by the dielectric spectra of the PECHs copolymers distinguish, at low temperatures, an intramolecular γ relaxation, which was associated with the reorientation of the benzyloxy substituent of the mesogenic side group. The addition of mesogenic side groups to the structure of PECHs does not affect the dynamic of the benzyloxy substituents. However, the crystalline organization, promoted by thermal treatment, increases the apparent activation energy of both copolymers.

At high temperatures, the intermolecular motions related to the glass (α_Tg_) transition were observed. The modification degree is the most relevant factor for α_Tg_ relaxations of both PECH copolymers. The presence of the dendritic side chains induces a crystalline order, which somewhat constrains the α_Tg_ relaxation process of the amorphous portions. Only when the crystalline order melts can the molecular motion of the backbone start, unlike other less complex semi-crystalline polymers. The dynamic fragility displays lower values for PECH80 copolymers than the PECH40 ones, and the self-organized columnar structure is more stable at temperatures below the glass transition. At temperatures near the glass transition, the crank-shaft movement promotes a quicker loosening of the ordered glass structure in PECH80 than in PECH40.

The α_Clear_ relaxation occurs near α_Tg_ relaxation and is related to a precursor motion of the disassembling of the polymeric columns. A higher number of the grafted dendritic group enhances the dispersion forces and the polymeric material turns isotropic faster.

In general terms, the possibility of designing PECH-based copolymers stable over a wide range of temperatures, depending on the number of the grafted dendritic group, allows fine-tuning the ionic transport properties of these membranes.

## Figures and Tables

**Table 1:** Calorimetric data for the investigated systems. Scan rate: 10 K/min.

Sample	T_m_ (K)	T_g_ (K)	T_c_ (K)	Δ*H_m_* (J/g)	ΔH_c_ (J/g)
PECH40	230–280	303	353	11.6	1.0
PECH80	225–290	306	403	17.3	1.9

**Table 2:** Dielectric strength of unoriented and oriented PECH80 and PECH40.

Δ ε″		**PECH80**		
	T (K)	153	163	173
	γ	0.030	0.032	0.028
	γ-O	0.022	0.023	0.024
	T (K)	223	233	243
	α_Tg_	0.094	0.149	0.245
	α_Tg_-O	0.050	0.059	0.080
	T (K)	343	353	363
	α_Clear_	0.218	0.232	0.246
	α_Clear_-O	0.079	0.112	0.127
Δ ε″		**PECH40**		
	T (K)	153	163	173
	γ	0.014	0.014	0.016
	γ-O	0.015	0.021	0.027
	T (K)	243	253	263
	α_Tg_	0.623	0.055	0.070
	α_Tg_-O	0.031	0.041	0.045
	T (K)	313	323	333
	α_Clear_	0.163	0.196	0.226
	α_Clear_-O	0.091	0.168	0.193

**Table 3:** Apparent activation energies of γ relaxation the gamma relaxation of the oriented and unoriented PECH copolymers.

Copolymer	*logf*_0_ (Hz)	*E_a_* (kJ·mol^−1^)	*T_max_* 1 kHz (K)	*R* ^2^
PECH80	13.7	26.5	129.1	0.99
PECH80-O	15.4	31.1	130.6	0.99
PECH40	14.9	30.2	132.7	0.99
PECH40-O	16.4	34.5	135.1	0.98

**Table 4:** VFTH parameters of α_Tg_ relaxation for unoriented and oriented PECH80 and PECH40 copolymers.

	T_g_ (K)	*τ*_0_ (s)	*D*	T_VFTH_ (K)	Φ_Tg_	α_Tg_ 10^4^ (K^−1^)	*R* ^2^
PECH80	287.0	8.1 ± 0.1	0.6 ± 0.1	237 ± 1	0.377	75.4	0.99
PECH80-O	283.3	7.9 ± 0.1	0.6 ± 0.1	233 ± 1	0.344	68.9	0.99
PECH40	270.8	9.7 ± 0.8	1.6 ± 0.5	220 ± 3	0.145	28.9	0.95
PECH40-O	270.8	9.7 ± 0.6	1.8 ± 0.3	221 ± 2	0.129	25.7	0.99

**Table 5:** VFTH parameters of α_Clear_ relaxation for unoriented and oriented PECH80 and PECH40.

α_Clear_	*τ*_0_ (s)	*D*	T_VFTH_ (K)	Φ_g_/B	*α_f_* × 10^4^ (K^−1^)	*R* ^2^
PECH80	10.4 ± 0.2	4.4 ± 0.18	260	0.044	8.7	0.98
PECH80-O	10.5 ± 0.4	4.4 ± 0.3	260	0.044	8.7	0.97
PECH40	8.5 ± 0.5	0.89 ± 0.12	---	---	---	0.92
PECH40-O	9.8 ± 0.7	1.9 ± 0.3	280	---	---	0.93

## Data Availability

The data presented in this study are available on request from the corresponding author. The data are not publicly available due to currently it forms part of an ongoing investigation.

## Supplementary Materials

The following are available online at https://www.mdpi.com/article/10.3390/polym13121961/s1, Figure S1. optical micrograph between crossed polars of PECH40, unoriented membrane after casting, room temperature.
