# Supplementary material for: Effect of Dendritic Side Groups on the Mobility of Modified Poly(epichlorohydrin) Copolymers
Source: Polymers (Basel). 2021 Jun 13;13(12):1961. doi: 10.3390/polym13121961 (PMC8231771; doi:10.3390/polym13121961)
Supplement: Supplementary file 1 [file polymers-13-01961-s001.zip › polymers-1246612-supplementary.pdf]

SUPPLEMENTARY MATERIALS

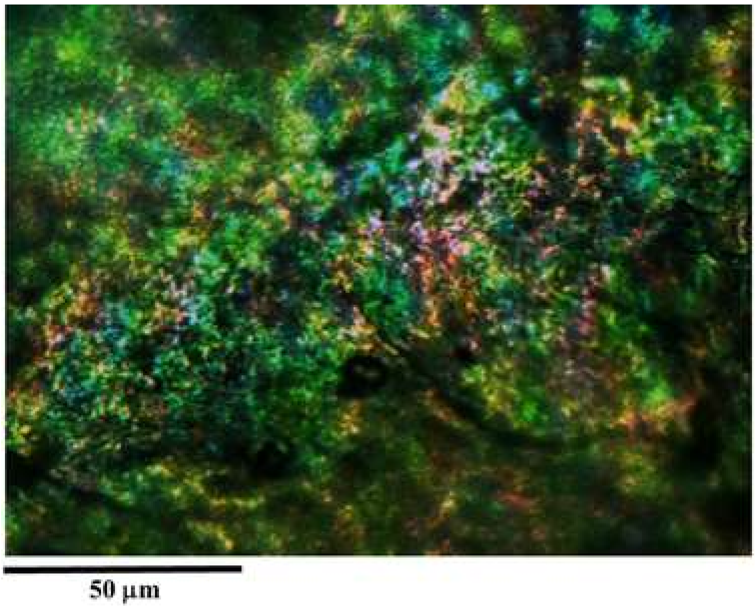

Figure S1 Optical micrograph between crossed polars of PECH40, unoriented membrane after casting, room temperature.
